# Do not let your guard down! Prevalence of *Dirofilaria immitis* and *Dirofilaria repens* in dogs entering shelters in northern Italy

**DOI:** 10.1186/s13071-025-07059-5

**Published:** 2025-10-14

**Authors:** Marco Genchi, Luigi Venco, Ornella Melideo, Laura Kramer, Marta Fozzer, Alice Vismarra

**Affiliations:** 1 Department of Veterinary Sciences, University of Parma, Strada del Taglio, 10, 43126 Parma, Italy; 2 Ospedale Veterinario Città di Pavia, Viale Cremona, 179, 27100 Pavia, Italy; 3 Elanco Italia, Via Ludovico di Breme, 13, 20156 Milano, Italy

**Keywords:** *Dirofilaria immitis*, *Dirofilaria repens*, Prevalence, Dogs, Northern Italy, Po River Valley

## Abstract

**Background:**

In Italy, the area of highest prevalence for canine heartworm disease (CHWD) has historically been along the Po River Valley in the northern area of the country, where prevalence in the mid-nineties ranged from 31% to 98%. Currently, increased awareness among practitioners and the availability of preventives have led to a dramatic decrease in prevalence in the area, although cases of CHWD continue to be diagnosed, suggesting the presence of canine reservoirs, including unowned dogs.

**Methods:**

The aim of the present study was to determine the prevalence of *Dirofilaria* spp. in stray dogs entering shelters located in the Po River Valley of northern Italy by using the modified Knott’s test and by antigenic testing.

**Results:**

Out of the 510 dogs tested, 173 (33.9%) were positive for circulating microfilariae: 15.7% (80/510) with *D. immitis*, 6.9% (35/510) with *D. repens*, and 11.4% (58/510) with a mixed infection.

**Conclusions:**

Unowned dog populations represent an important reservoir for the parasite, allowing *D. immitis* to remain in a given geographical area and increasing the risk of exposure to all dogs.

**Graphical Abstract:**

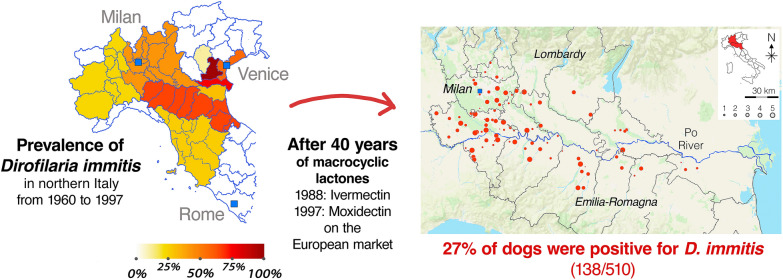

## Background

*Dirofilaria immitis*, the agent of canine heartworm disease (CHWD), was first described in 1626 by Francesco Birago, an Italian nobleman who reported finding worms in the heart of one of his hunting dogs [1]. Four hundred years later, *D. immitis* is known to be present around the world and is endemic in several European countries, including Italy [2].

Prevention of CHWD is based on the larvicidal activity of macrocyclic lactones (MLs), which eliminate migrating L3–L4 larvae before they can reach maturity [3]. In 1982, the efficacy of ivermectin against *Dirofilaria immitis* larvae and in preventing development of adult heartworms was first reported [4]. In 1987, it was introduced as a monthly heartworm preventive in Europe. There is currently a wide range of macrocyclic lactones in various formulations registered in Europe for the prevention of CHWD [5].

In Italy, the area of highest prevalence for CHWD has historically been along the Po River Valley in the northern area of the country [6], where prevalence in the mid-nineties ranged from 31% to 98% [7]. Currently, increased awareness among practitioners and the availability of preventives have led to a dramatic decrease in prevalence in the area [8]. On the other hand, in areas of Italy where awareness is lower and prevention is recommended less often, prevalence in dogs is increasing [9]. Interestingly, two recent surveys reported that veterinary practitioners in northern Italy continue to diagnose the infection in dogs, with practices from several provinces reporting over 20 cases of infection in the 12 months preceding the surveys [10, 11]. This would suggest that *D. immitis* continues to circulate in northern Italy, despite the same surveys reporting that over 95% of practitioners recommend prevention. Numerous studies have shown that heartworm prevalence is higher in dogs that generally lack veterinary care. Gettings et al. [12] recently reported that *D. immitis* infection prevalence was higher in shelter-housed dogs compared with owned dogs. An earlier study showed that heartworm prevalence in shelter dogs was over 10% higher than in owned dogs [13]. Studies from southern Italy indicate very high prevalence in shelters, ranging from 44% to 75% [14, 15].

The first description of *Dirofilaria repens*, the agent of canine and human subcutaneous dirofilariosis (SCD), was likely reported in 1566 by a Portuguese physician who observed the parasite in the eye of a 3-year-old girl [16]. *D. repens* is currently endemic in several different European countries and is considered an emerging zoonosis, with over 3500 human cases reported in Europe from 1977 to 2016 [17]. The most recent data indicate that *D. repens* is practically endemic throughout Italy, with prevalence in dogs ranging from 1.5% to 12% [18]. In two recent surveys, practitioners working in the Po River Valley reported diagnosing between 1 and 20 cases of canine SCD in the 12 months preceding the survey [10, 11]. Infection in dogs is frequently asymptomatic, and only a few reports of clinically manifested disease have been reported [19]. Prevention of canine SCD is also based on the larvicidal activity of macrocyclic lactones. However not all MLs used for the prevention of CHWD are effective against *D. repens* [20].

Stray dog populations represent an important reservoir for *D. immitis* and *D. repens*, allowing the parasites to circulate in a given geographical area, with a high density and wide distribution of mosquito populations, and to increase the risk of exposure to all dogs.

The aim of the present study was to determine the prevalence of *D. immitis* and *D. repens* in stray dogs entering shelters located in the Po River Valley of northern Italy.

## Methods

### Animals

Dogs admitted to municipal dog shelters in 13 provinces of the Lombardy and Emilia-Romagna regions of the Po River Valley from January 2024 to January 2025 were included in the study. Only unowned, stray dogs with no means of identification (i.e., microchip) with an estimated age of > 10 months were tested. Size (small, medium, large, and giant), sex, and estimated age were recorded for each dog entering the shelters. Dogs from 10 months to 3 years of age were considered “young,” from 4 to 6 years “adult,” and over 7 years “old.” Clinical examination was carried out by the shelter practitioner, including auscultation of heart and lungs and the presence/absence of cough/ascites. Two milliliters of whole blood in EDTA was collected from each dog.

### Knott’s test

Knott’s test was carried out according to Bazzocchi et al. [21], modified according to Ref. [22]. Briefly, 1 ml of blood in EDTA was mixed with 9 ml of distilled water in a 15-ml tube. The tube was gently inverted several times to mix the solution and then centrifuged for 3 min at 1500 × *g*. The supernatant was poured off, and 100 µl of sediment was mixed with 100 µl of 1% methylene blue stain. Twenty microliters of stained sediment was placed on a slide, covered with a coverslip, and examined under a microscope. Species identification of all microfilariae (mff) in the sediment was carried out by measuring the length and evaluating the head and tail, according to Ref. [20]. The number of mff (of both species in the case of mixed infection) was multiplied by 50 and is expressed as mff/ml.

### Antigen testing

Antigen testing was carried out using whole blood. The commercial kit SNAP^®^ 4Dx^®^ Plus (IDEXX Laboratories Inc., Westbrook, Maine, USA) was used according to the manufacturer’s instructions.

The results obtained from Knott’s tests and the serological tests were promptly communicated to the veterinarian responsible for the shelters.

### Statistical analyses

To evaluate whether specific factors or combinations of multiple factors (e.g., sex, size, and age) may be associated with the presence of infection, data were compared by one-way analysis of variance (ANOVA) test and a Student’s *T* test using GraphPad Prism^®^ version 10.5.0 software (GraphPad Software, San Diego, CA, USA). *P*-value lower than 0.05 was considered statistically significant.

## Results

**Fig. 1:**
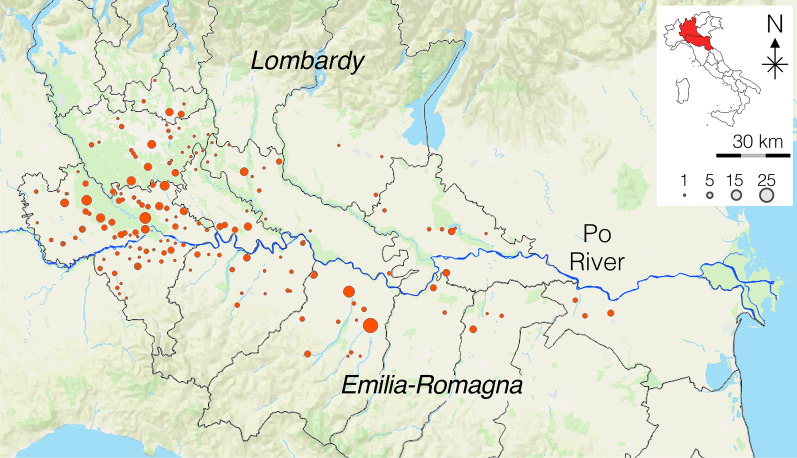
Geographical localization of 510 tested dogs

A total of 510 dogs from the Lombardy and Emilia-Romagna regions were tested (Fig. 1). Table 1 reports demographics of the study population.

**Table 1:** Demographic data of 510 dogs analyzed in the study

	Sex		Age			Size			
	Male (%)	Female (%)	Young (%)	Adult (%)	Old (%)	Small (%)	Medium (%)	Large (%)	Giant (%)
Total	300 (58.8)	210 (41.2)	92 (18.0)	178 (34.9)	240 (47.1)	173 (33.9)	239 (46.9)	88 (17.3)	10 (2.0)

**Fig. 2:**
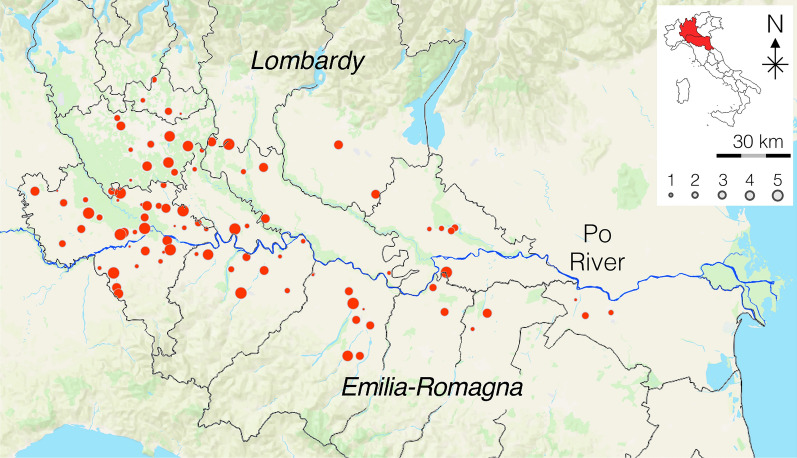
Dogs positive for *D. immitis* (modified Knott’s test)

**Fig. 3:**
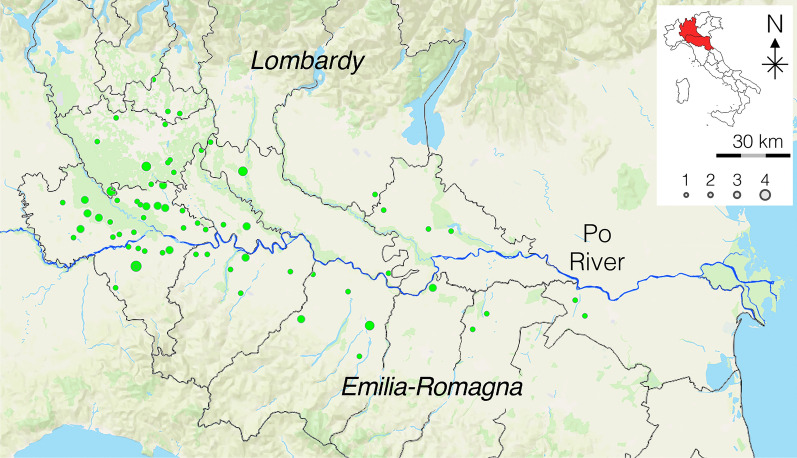
Dogs positive for *D. repens* (modified Knott’s test)

**Fig. 4:**
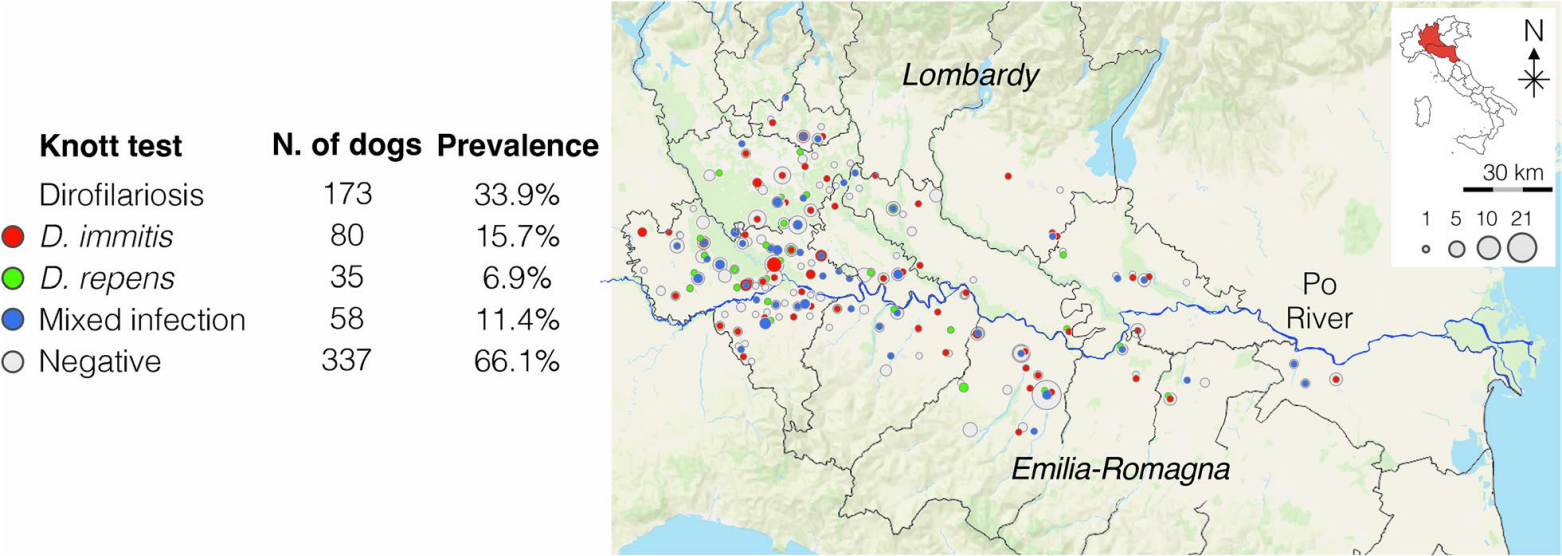
Dogs positive and negative for dirofilariosis tested by Knott’s test. Red: *D. immitis*; green: *D. repens*; blue: mixed infection; grey: negative

Table 2 summarizes the results of the modified Knott’s and antigen testing. One hundred seventy-three dogs (33.9%) were positive for circulating mff: 15.7% (80/510) with *D. immitis* (Fig. 2), 6.9% (35/510) with *Dirofilaria repens* (Fig. 3), and 11.4% (58/510) with a mixed infection (Fig. 4). The antigenic prevalence was 30.6% (156/510). Twenty-four (4.7%) dogs were antigen-positive but microfilariae-negative. Six (1.2%) dogs were microfilaremic for *D. immitis* but antigen negative. Microfilaremia ranged from 50 to 2980/ml for *Dirofilaria immitis* and from 5 to 1250/ml for *Dirofilaria repens*. Eight (22.9%) of the 35 dogs with *D. repens* mff were also positive for *D. immitis* antigens. Of those dogs positive for *D. immitis*, 2.5% presented with cough and 1.0% presented with ascites.

**Table 2:** Demographic data of the dogs infected with *D. immitis*, *D. repens*, or both

	Sex		Age			Size			
	Male (%)	Female (%)	Young (%)	Adult (%)	Old (%)	Small (%)	Medium (%)	Large (%)	Giant (%)
Negative	206 (68.7)	131 (62.4)	74 (80.4)	106 (59.6)	157 (65.4)	126 (72.8)	159 (66.5)	44 (50.0)	8 (80.0)
Positive (*Dirofilaria* spp.)	94 (31.3)	79 (37.6)	18 (19.6)	72 (40.5)	83 (34.6)	47 (27.2)	80 (33.5)	44 (50.0)	2 (20.0)
*D. immitis*	45 (15.0)	35 (16.7)	4 (4.4)	32 (18.0)	44 (18.3)	21 (12.1)	33 (13.8)	26 (29.6)	0 (0.0)
*D. repens*	20 (6.7)	14 (7.1)	7 (7.6)	15 (8.4)	13 (5.4)	11 (6.4)	19 (8.0)	5 (5.7)	0 (0.0)
Mixed infection	29 (9.7)	29 (13.8)	7 (7.6)	25 (14.0)	26 (10.8)	15 (8.7)	28 (11.7)	13 (14.8)	2 (20.0)

Statistical analyses highlighted a significant difference for prevalence of *D. immitis*, *D. repens*, or both (*P* = 0.04*), with higher prevalence in adult/old dogs compared with young dogs. Medium-sized dogs also had significantly higher prevalence compared with others (*P* = 0.022*).

## Discussion

The use of macrocyclic lactones for the prevention of CHWD is widely applied in northern Italy and has led to a dramatic decrease in prevalence of disease among owned dogs that are under the care of veterinary practitioners [10, 11].

Even though ivermectin was introduced onto the Italian market in 1987, followed by other MLs, the prevalence of CHWD was still high in the mid-nineties in the historically endemic area of the Po River Valley. According to more recent studies, however, prevalence in owned dogs in northern Italy is reported as ranging from 0% to 6% [23–25]. This suggests that it took several decades for widespread use of MLs for prevention to become established practice. The results of the present study, however, confirm that *D. immitis* continues to circulate in dogs in the Po River Valley that are not receiving veterinary care and that are likely not receiving prevention with MLs. As previously reported by others, in the present study, dogs over 3 years of age had higher prevalence of *Dirofilaria* spp. infection compared with young dogs [6]. Significantly higher risk of infection was also observed in larger-sized dogs.

The results of this study also highlight an important prevalence for *D. repens*, the agent of subcutaneous dirofilariosis and an emerging zoonosis of concern [26]. There is much less information regarding the true prevalence of *D. repens* in dogs from the Po River Valley: it is likely underdiagnosed, due to the lack of a specific antigen test and the subclinical nature of the infection. Not all available MLs are effective in preventing *D. repens* in dogs (the main reservoir) [20], and our results suggest that practitioners should recommend preventives that are efficacious for both. Furthermore, it would be beneficial to recommend other types of testing for *D. repens* such as conventional polymerase chain reaction (PCR) or quantitative Real-time PCR (qPCR) to detect DNA of microfilariae within dog blood [27]. Note that 7 of 35 dogs positive only for *D. repens* mff were also positive on heartworm antigen testing, indicating either occult heartworm infection or cross-reactivity, as reported previously [28].

Even though all *Dirofilaria* spp.positive dogs were sourced locally, there is no way of knowing where they originated from. Indeed, many rescue operations bring dogs into the area from central and southern Italy (areas of high prevalence), which may then be abandoned or lost [29]. So, we cannot be sure that the infection was contracted in the study area. However, Vismarra et al. [30] recently reported that *D. immitis* also continues to circulate in mosquitoes in several areas of the Po River Valley, suggesting stable endemicity and risk of infection in unprotected dogs.

## Conclusions

*D. immitis* continues to circulate in the Po River Valley. This is important for veterinary practitioners, dog owners, and shelter staff. Routine screening of all dogs entering shelters with Knott’s and antigen testing, as required by the regional veterinary health services, is a unique opportunity to understand the prevalence of *D. immitis* and *D. repens* prior to clinical signs or prior to advanced disease progression and to reduce the risk of zoonotic transmission.

## Data Availability

No datasets were generated or analyzed during the current study. All the data are presented herein.

## Abbreviations

*D. immitis*: Dirofilaria immitis
*D. repens*: Dirofilaria repens
CHWD: Canine heartworm disease
mff: Microfilariae
L2–L3: Second-stage and third-stage larvae
SCD: Subcutaneous dirofilariosis
MLs: Macrocyclic lactones
EDTA: Ethylenediaminetetraacetic acid
